# Kinetochore protein MAD1 participates in the DNA damage response through ataxia-telangiectasia mutated kinase-mediated phosphorylation and enhanced interaction with KU80

**DOI:** 10.20892/j.issn.2095-3941.2020.0044

**Published:** 2020-08-15

**Authors:** Mingming Xiao, Xuesong Li, Yang Su, Zhuang Liu, Yamei Han, Shuai Wang, Qinghua Zeng, Hong Liu, Jianwei Hao, Bo Xu

**Affiliations:** ^1^Department of Biochemistry and Molecular Biology, Key Laboratory of Breast Cancer Prevention and Therapy, Ministry of Education, Tianjin Medical University Cancer Institute and Hospital, National Clinical Research Center for Cancer, Key Laboratory of Cancer Prevention and Therapy, Tianjin, Tianjin’s Clinical Research Center for Cancer, Tianjin 300060, China; ^2^Department of Oncology, Southern Research Institute, Birmingham, AL 35205, USA; ^3^Department of Neurosurgery, Tianjin Huanhu Hospital, Tianjin 300350, China; ^4^Tianjin Key Laboratory of Cerebral Vascular and Neurodegenerative Diseases, Tianjin 300350, China; ^5^Center for Intelligent Oncology, Chongqing University Cancer Hospital, Chongqing University School of Medicine, Chongqing 400030, China

**Keywords:** DNA damage response, ataxia-telangiectasia mutated kinase (ATM), mitotic arrest-deficient protein 1 (MAD1), KU80 protein, DNA-PKcs

## Abstract

**Objective:** Mitotic arrest-deficient protein 1 (MAD1) is a kinetochore protein essential for the mitotic spindle checkpoint. Proteomic studies have indicated that MAD1 is a component of the DNA damage response (DDR) pathway. However, whether and how MAD1 might be directly involved in the DDR is largely unknown.

**Methods:** We ectopically expressed the wild type, or a phosphorylation-site--mutated form of MAD1 in MAD1 knockdown cells to look for complementation effects. We used the comet assay, colony formation assay, immunofluorescence staining, and flow cytometry to assess the DDR, radiosensitivity, and the G2/M checkpoint. We employed co-immunoprecipitation followed by mass spectrometry to identify MAD1 interacting proteins. Data were analyzed using the unpaired Student’s *t-*test.

**Results:** We showed that MAD1 was required for an optimal DDR, as knocking down MAD1 resulted in impaired DNA repair and hypersensitivity to ionizing radiation (IR). We found that IR-induced serine 214 phosphorylation was ataxia-telangiectasia mutated (ATM) kinase-dependent. Mutation of serine 214 to alanine failed to rescue the phenotypes of MAD1 knockdown cells in response to IR. Using mass spectrometry, we identified a protein complex mediated by MAD1 serine 214 phosphorylation in response to IR. Among them, we showed that KU80 was a key protein that displayed enhanced interaction with MAD1 after DNA damage. Finally, we showed that MAD1 interaction with KU80 required serine 214 phosphorylation, and it was essential for activation of DNA protein kinases catalytic subunit (DNA-PKcs).

**Conclusions:** MAD1 serine 214 phosphorylation mediated by ATM kinase in response to IR was required for the interaction with KU80 and activation of DNA-PKCs.

## Introduction

An optimal DNA damage response (DDR), in dealing with a variety of exogenous or endogenous DNA damage insults, is essential for the organism to preserve the stability and integrity of its genome^1^. The functional significance of the DDR in prevention of tumorigenesis has been well documented^2,3^. The DDR processes range from transcriptional regulation to post-translational modifications (e.g., phosphorylation, acetylation, methylation, ubiquitination, and SUMOylation). Key elements involved in the process include damage sensors, transducers, and mediators. Sensors, such as chromatin-bound proteins, detect DNA lesions and recruit transducers. Transducers, typically protein kinases, amplify signals by modification of effectors in the presence of mediators. Effectors then initiate cell cycle checkpoints and DNA repair. The ataxia-telangiectasia mutated (ATM) kinase is a central protein critical in the transduction of the messages in response to double-stranded breaks^4^. Mutation of the *ATM* gene causes the autosomal recessive disorder, ataxia telangiectasia (A-T). A-T patients are characterized by progressive neurodegeneration, immunodeficiency, cancer predisposition, and a hypersensitivity to ionizing radiation (IR)^5^. A large number of proteins can be phosphorylated by ATM kinase in response to DNA damage^6^.

Another mechanism used to guard against chromosomal instability is the mitotic spindle assembly checkpoint (SAC), which ensures proper chromosome segregation during cell division^7,8^. The activation of the SAC requires involvement of kinetochore proteins.

The kinetochore is a nuclear structure consisting of a protein complex that binds to chromatids, allowing spindle formation required for sister chromatid segregation during mitosis. This protein structure can be divided into inner plates as a part of the chromatin structure, and outer plates, which contain more than 20 proteins. Proteins in the outer plates are essential elements for activation of the SAC. Mitotic arrest-deficient protein 1 (MAD1), MAD2, MAD3, MPS1, BUB1, and BUB3 are core components of the SAC. MAD1 recruits MAD2 to assemble the mitotic checkpoint complex. Once MAD1 is localized to the kinetochore, the SAC is fully activated^9^. Altered (either upregulated or downregulated) expression of MAD1 has been reported in various cancers, suggesting its involvement in tumorigenesis. For example, upregulation of MAD1 can lead to the wrong localization of MAD2, causing SAC defects and chromosomal instability^10^. MAD1 is also frequently upregulated at both the mRNA and protein levels in human breast cancers, where it serves as a marker of poor prognosis^11^. However, reduced MAD1 expression is linked to the increased rate of cancer in mice^12^.

Due to their overlapping functions in the maintenance of genomic stability, it is believed, and evidence has proven, that the DDR and SAC networks have overlapping protein functions. For example, DDR elements ATM^13^, BRCA1^14–16^, BRCA2^17^, CHK1^18^, and CHK2^14–16^ are involved in SAC regulation, and BUB1, BUBR1, and MAD2 kinetochore proteins have been reported to participate in the DDR^8,19–21^. We previously showed that MAD1 was linked to the mitotically activated ATM kinase^22^. In mitosis, ATM kinase phosphorylates MAD1 on serine 214 to regulate the SAC^22^.

Notably, MAD1 is one of the four mitotic proteins initially identified by mass spectrometry as an ATM kinase substrate in response to IR^6^, suggesting that a link between the ATM kinase to MAD1 might be essential for the DDR. In the current report, we showed a critical role for MAD1 in dealing with IR-induced DNA damage through ATM kinase-mediated phosphorylation and interaction with KU80 and the DNA protein kinase catalytic subunit (DNA-PKcs).

## Materials and methods

### Cell lines and culture

The HeLa human cervical cancer cell line and HCT116 colorectal cancer cell line (The American Type Culture Collection, Manassas, VA, USA) were maintained in Dulbecco’s Modified Eagle’s Medium (DMEM) (for HeLa) or Roswell Park Memorial Institute (RPMI) 1640 medium (for HCT116) containing 10% fetal bovine serum, supplemented with 4 mM of L-glutamine and 50 µg/mL of penicillin/streptomycin (all from HyClone Laboratories, Logan, UT, USA). The cells were cultured in an incubator with 5% CO_2_ at 37 °C. The isogenic cell lines expressing control or ATM kinase shRNA were maintained in DMEM supplemented with 1 µg/mL puromycin. The simian virus 40-transformed human fibroblast cell lines, GM0637 and GM9607 (The NIGMS Human Mutant Cell Repository, Camden, NJ, USA) were maintained in RPMI1640 medium supplemented with 10% fetal bovine serum and 50 µg/mL of penicillin/streptomycin.

### Irradiation

We used an X-Rad 320 irradiator (Precision X-Ray, Branford, CT, USA) at a dose rate of 2 Gy/min (*in vitro* studies) and 6 MV X-ray irradiation (600CD; Varian, Palo Alto, CA, USA) at a dose rate of 4 Gy/minute of delivered IR (*in vitro* and *in vivo* studies).

### Plasmids, antibodies, and reagents

To generate FLAG-tagged MAD1, the full-length coding sequence of MAD1 was obtained by RT-PCR and subcloned into the pcDNA3.1 vector at the BamHI-XbaI sites, with the primers as following: 5′-ACTGGATCCACGATGATGGACTACAAGGACGATGACGACAAGATGGAAGACCTGGGGAAAACACCA-3′ and 5′-AGCTCTAGACTACGCCACGGTCTGGCGGCTGAAGAG-3′. The MAD1-S214A mutant was generated using the QuikChange II XL Site-directed Mutagenesis Kit (Stratagene, San Diego, CA, USA) according to the manufacturer’s protocol. The primers used were 5′-GAACTCCAGGCCGCACAAGAAGCAAGAGCAGACCACGAGCAGC-3′ and 5′-GCTGCTCGTGGTCTGCTCTTGCTTCTTGTGCGGCCTGGAGTTC-3′. The rabbit phosphor-MAD1-S214 antibodies were raised against the KIQELQApSQEARA-NH_2_ peptide by Abgent (San Diego, CA, USA). Anti-MAD1, anti-γH2AX, and anti-DNA PK were obtained from Abcam (Cambridge, MA, USA). The anti-FLAG antibody was obtained from OriGENE (Rockville, MD, USA), and anti-histone-H3-Ser10p was from Millipore (Billerica, MA, USA).

### Immunofluorescence microscopy

For immunofluorescence microscopy experiments, we fixed cells in 4% paraformaldehyde for 15 min at room temperature and blocked them with 1% bovine serum albumin in phosphate-buffered saline (PBS) for 30 min. Antibodies against MAD1 S214p, and γH2AX were incubated at 4 °C overnight. After washing three times with PBS, the cells were then incubated with fluorescence-conjugated secondary antibodies for 1 h. After washing three more times with PBS, 4′,6-diamidino-2-phenylindole (0.5 µg/mL) was added to stain the cell nuclei. The coverslips were then mounted onto glass slides and subjected to microscopy using a DP71 microscope (Olympus, Tokyo, Japan).

### Flow cytometry

The cells were harvested and fixed in 70% ethanol and permeabilizd with 0.1% Triton X-100 on ice for 5 min, followed by blocking in PBS with 1% bovine serum albumin for 30 min. The fixed cells were then incubated with anti-phospho-histone-H3-Ser10 for 1 h at room temperature and the fluorescein isothiocyanate-conjugated secondary antibody for 60 min in the dark. The cells were then stained with 25 µg/mL propidium iodide (Life Technology, Grand Island, NY, USA) and the percentage of mitotic cells was quantified using a FACS Calibur flow cytometer (Becton Dickinson, Franklin Lakes, NJ, USA) with CellQuest software.

### Immunoprecipitation and Western blot

For immunoprecipitation, whole cell lysates were incubated with anti-FLAG-M2 agarose beads (Sigma-Aldrich, St. Louis, MO, USA) overnight at 4 °C, washed three times with 500 µL TBS buffer, and then eluted using 80 µL Laemmli buffer (2% SDS, 10% glycerol, 5% DTT, and 0.125 M Tris-HCl, pH = 6.8). The eluted proteins were analyzed on an SDS-PAGE gel followed by probing with various antibodies. For general Western blot analyses, protein samples were separated by SDS-PAGE and transferred to nitrocellulose membranes. After incubating with primary and secondary antibodies, the signals were detected using a chemiluminescence detection system (Millipore, Burlington, MA, USA).

### In-gel digestion and liquid chromatography-mass spectrometry/mass spectrometry

Gels were first visualized using a silver staining kit based on the manufacturer’s protocol (Thermo Fisher Scientific, Waltham, MA, USA). Each gel lane was divided into 8 fractions and digested with trypsin before mass spectrometric analysis. The normal IgG control samples were then analyzed in parallel to distinguish the nonspecific binding proteins. Tryptic peptides were analyzed using a nanoUPLC-ESI QE plus mass spectrometer (Thermo Fisher Scientific).

### Colony formation assay

Radiation sensitivity was measured by the colony formation assay. Exponentially growing HeLa and HCT116 cells were plated into 6-well plates 1 day before they were irradiated (0–6 Gy). After 2 week incubation, surviving colonies were fixed with 20% of methanol and stained with Crystal Violet before the number of surviving colonies (> 50 cells) was counted.

### The comet assay

The cells were irradiated with 0 or 6 Gy and incubated for 2 h. Cells were then collected and processed according to the manufacturer’s protocol (CometAssay 4250-050-K, Trevigen, Gaithersburg, MD, USA).

### Xenografts in nude mice

Four-week-old female BALB/c nu/nu mice (Beijing Experimental Animal Center, Beijing, China) were used for *in vivo* studies. All animal studies were performed following the animal procedures approved by the Institutional Animal Care and Use Committee of Tianjin Medical University Cancer Institute and Hospital, which is consistent with national regulatory standards. A total of 5 × 10^6^ HCT116 cells stably expressing either control shRNA, MAD1 shRNA, MAD1shRNA + WT, or MAD1shRNA + S214A were injected into the right thigh of nude mice. When the average tumor volume reached 300 mm^3^, we irradiated the xenografts with 0 or 10 Gy. Two-dimensional tumor sizes were measured using a caliper once every 3 days during 30 days. The tumor volume was calculated using the formula: volume = width^2^ × length × 0.5. The growth delay rate = (t × log_2_)/[log (V_t_/V_0_)], where “t” represented the interval between two measurements, and V_t_ and V_0_ represented xenograph volumes for the initial and last measurements, respectively.

### Statistical analysis

All data for statistical analyses were obtained from at least 3 independent experiments. Statistical analyses were performed using Prism software, version 7.0 (GraphPad, San Diego, CA, USA). The unpaired Student’s *t-*test was used for the significance analysis. Values of *P* ≤ 0.05 were considered to be significant.

## Results

### MAD1 is required for an optimal DNA damage response

**Figure 1:**
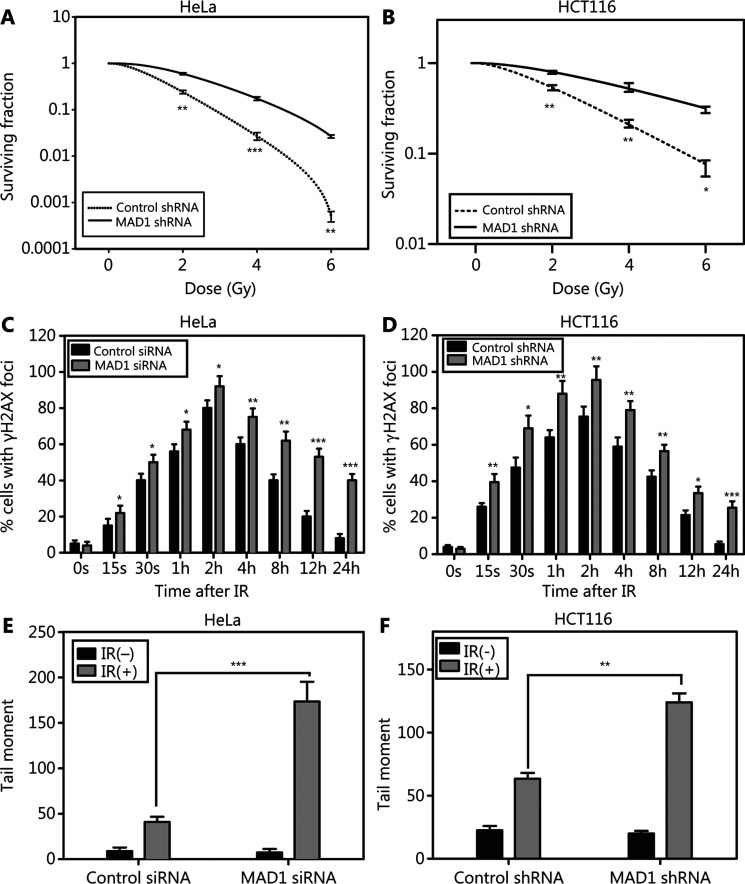
Mitotic arrest-deficient protein 1 (MAD1) is required for the DNA damage response. Control siRNA or pooled MAD1 siRNA oligonucleotides were transiently transfected into HeLa cells, or control shRNA or MAD1 shRNA were stably transfected into HeLa or HCT116 cells. (A and B) Cellular radiosensitivity was assessed by the colony formation assay in isogenic cell lines after ionizing radiation (IR) (0–6 Gy). Shown are the averages of at least triplicate samples. Standard errors are shown by error bars. (*P < 0.05; **P < 0.01; ***P < 0.001) (C and D). Nuclear γH2AX foci formation were counted in cells and shown as the averages of 100 cells. Standard errors are shown by error bars. Statistical analyses were conducted using Student’s *t*-test (*P < 0.05; **P < 0.01; ***P < 0.001). (E and F) Olive Tail Moment was recorded in the single cell gel electrophoresis assay. Shown are the averages of 100 cells. Standard errors are shown by error bars (*P < 0.05; **P < 0.01; ***P < 0.001).

Because MAD1 is a downstream target of ATM kinase in mitosis^22^, and it is one of the four targets identified by a proteomics study^6^, we determined whether MAD1 might also be directly involved in the DDR. To achieve this goal, we knocked down MAD1 using siRNA or shRNA. Reduction of MAD1 expression resulted in a reduced cellular survival after IR. With a pooled siRNA knockdown (**Supplementary Figure S1A**) in HeLa cells, the cells became hypersensitive to IR (**Figure 1A**). Similarly, downregulation of MAD1 in HCT116 cells showed hypersensitivity to IR (**Supplementary Figure S1B and Figure 1B**). Using fluorescence microscopy, we found that HeLa and HCT116 cells with depleted MAD1 displayed a prolonged existence of γH2AX foci (**Figure 1C and 1D**), indicating an abnormality in DNA damage repair. We also conducted single cell gel electrophoresis (COMET) to assess DNA fragmentation after IR. We found that with MAD1 knockdown, both HeLa and HCT116 cells displayed a significant increase in the comet tail after IR, when compared to control cells (**Figure 1E, 1F, and Supplementary Figure S2A**), indicating that DNA strand break repair was significantly impaired when MAD1 was knocked down. Taken together, these results demonstrated that MAD1 was required for an optimal DDR.

### IR-induced MAD1 serine 214 phosphorylation is ATM kinase-dependent

**Figure 2:**
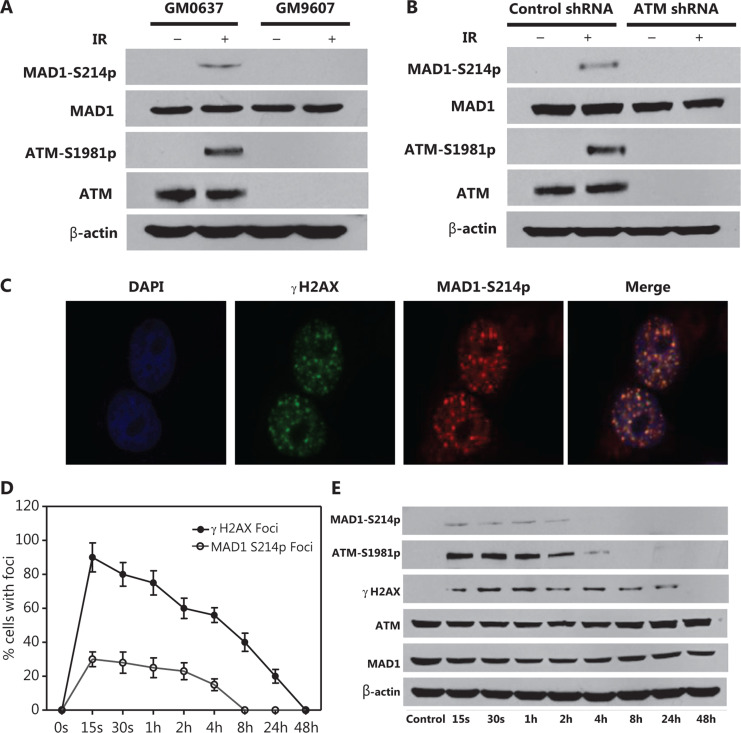
Mitotic arrest-deficient protein 1 (MAD1) serine 214 phosphorylation is ataxia-telangiectasia mutated (ATM) kinase-dependent and the protein forms at nuclear foci. (A) SV-40 transformed fibroblast cell lines GM0637 (ATM^+/−^) and GM9607 (ATM^−/−^) were treated with mock or irradiated radiation [ionizing radiation (IR); 6 Gy], and (B) HeLa cells stably transfected with control or ATM kinase shRNA were treated with mock or IR (6 Gy). Two hours after IR, whole cell lysates were harvested and subjected to immunoblotting using the indicated antibodies. (C) HeLa cells were mock treated or irradiated for 6 Gy. At different time points after IR, cells were subjected to immunofluorescence microcopy using the indicated antibodies. The above images are from cells 2 h after IR. (D) Nuclear γH2AX foci were counted and shown as the averages of 100 cells. Standard deviations are shown by error bars. (E) HeLa cells were treated with mock or IR (6 Gy). At different time points after IR, total cell lysates were harvested and immunoblotted with the indicated antibodies.

To test potential MAD1 phosphorylation by ATM kinase in response to IR, we used a polyclonal phosphor-specific antibody recognizing MAD1 phosphorylation on serine 214, which we had previously generated^22^. Using this antibody, we assessed MAD1 serine 214 phosphorylation in SV-40 transformed fibroblast cell lines with proficient (GM0637) or deficient (GM9607) ATM kinase after mock-treatment or irradiation (5 Gy). **Figure 2A** shows that GM0637 cells showed a MAD1 serine 214 phosphorylation signal when exposed to IR. In contrast, GM9607 cells did not show a detectable IR-induced MAD1 serine 214 phosphorylation, suggesting a role of ATM kinase in this process. To further test the ATM kinase dependency, we used a pair of isogenic HeLa cell lines in which ATM kinase was stably transfected with control or ATM kinase shRNA^13^. We found that control shRNA cells displayed a strong phosphorylation signal in response to IR. However, MAD1 serine 214 phosphorylation was deficient in ATM kinase-shRNA knockdown cells (**Figure 2B**), demonstrating that ATM kinase -dependent MAD1 serine 214 phosphorylation was induced by IR.

To investigate the impact of ATM kinase-mediated MAD1 serine 214 phosphorylation on cell cycle checkpoints, we measured the cell cycle with propidium iodide and a phosphor-histone H3 antibody, which used a mitotic marker that indicated the G2 to M transition. We found that MAD1 shRNA and the MAD1 S214A mutation had no effect on the G2/M checkpoint (**Supplementary Figure S3A**). We also synchronized HeLa cells via double thymidine blockage, and found that regardless of cell cycle stages, serine 214 phosphorylation was induced after IR. This result indicated that IR-induced MAD1 serine 214 phosphorylation was independent of cell cycle stages (**Supplementary Figure S3B**).

Using immunofluorescence microscopy, we also observed that MAD1 serine 214 phosphorylation caused nuclear foci in response to IR (**Figure 2C**), and MAD1 foci showed a co-localization pattern with the γH2AX foci (**Figure 2C**). Similar to γH2AX and ATM kinase phosphorylation, MAD1 serine 214 phosphorylation was shown in a time-dependent manner (**Figure 2D and 2E**). Together, the data indicated a spatial and temporal pattern of serine 214 phosphorylation.

### ATM kinase-mediated MAD1 serine 214 phosphorylation is required for an optimal DDR

**Figure 3:**
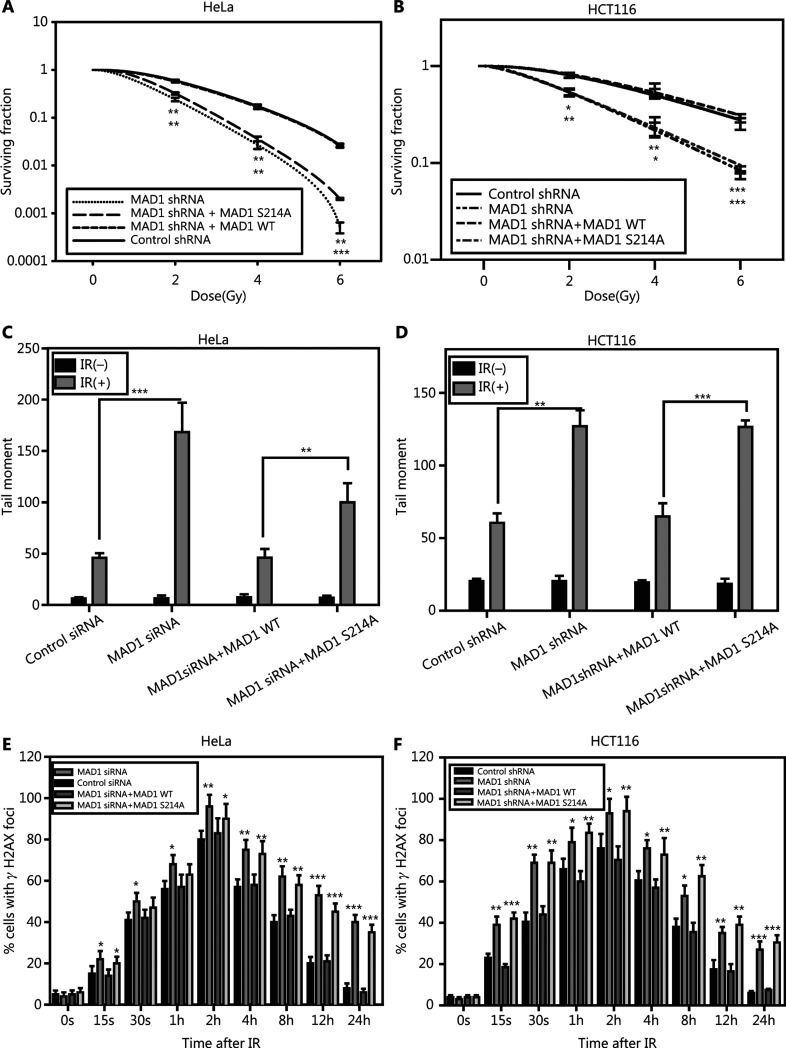
Ataxia-telangiectasia mutated (ATM) kinase-mediated mitotic arrest-deficient protein 1 (MAD1) serine214 phosphorylation is required for the DNA damage response. Control or MAD1 knockdown cells were transiently transfected with the siRNA-resistant wild type or S214A mutant of MAD1 in HeLa or HCT116 cells. (A and B) Cellular radiosensitivity was assessed by the colony formation assay in isogenic cell lines after irradiation (0–6 Gy). Shown are the averages of at least triplicate samples. Standard errors are shown by error bars (*P < 0.05; **P < 0.01; ***P < 0.001). (C and D) Olive Tail Moment was recorded in the single cell gel electrophoresis assay. Shown are the averages of 100 cells. Standard errors are shown by error bars. Statistical analyses were conducted using Student’s t-test (*P < 0.05; **P < 0.01; ***P < 0.001). (E and F) Nuclear γH2AX foci formation were counted in each cell and shown as the average of 100 cells. Standard errors are shown by error bars (*P < 0.05; **P < 0.01; ***P < 0.001).

To study the functional significance of IR-induced MAD1 serine 214 phosphorylation, we generated siRNA or shRNA resistant MAD1 constructs (wild type or serine 214 mutated to alanine, S214A, which could no longer be phosphorylated at the site) and expressed the constructs into MAD1 siRNA knockdown HeLa cells and MAD1 shRNA knockdown HCT116 cells (**Supplementary Figure S1B and S1C**). We found that wild type MAD1 expression in MAD1 knockdown cells rescued the radiosensitive phenotype, when using the colony formation assay (**Figure 3A and 3B**). However, the S214A mutant of MAD1 failed to do so. Similarly, the S214A mutant resulted in an enhanced comet tail moment (**Figure 3C, 3D and Supplementary Figure S2B**) and it could not complement the prolonged γH2AX foci in MAD1 knockdown cells (**Figure 3E and 3F**). Taken together, these results provided strong evidence that MAD1 serine 214 phosphorylation was critical for the optimal DNA damage response after IR.

### MAD1 forms a protein complex in response to IR-induced DNA damage

To further study the functional significance of MAD1 serine 214 phosphorylation in the DDR, we aimed to identify a potential MAD1 complex that was associated with IR-induced phosphorylation. To achieve this goal, we transfected FLAG-tagged wild type or the S214A mutant of MAD1 into HeLa cells. Then, we mock treated or irradiated the cells, and immunoprecipitated the FLAG-tagged proteins (**Supplementary Figure S4**). We compared the difference between the IR response in wild type or S214A expressing cells. A total of 5 bands were recognized with alterations before and after IR, which were significant in wild type and S214A cells. These bands in the silver stained gels were removed and sent to a mass spectrometry facility for protein identification. The list of proteins identified are shown in **Supplementary Table S1**.

### ATM kinase-mediated MAD1 phosphorylation is required for an IR-induced MAD1-KU80 interaction

**Figure 4:**
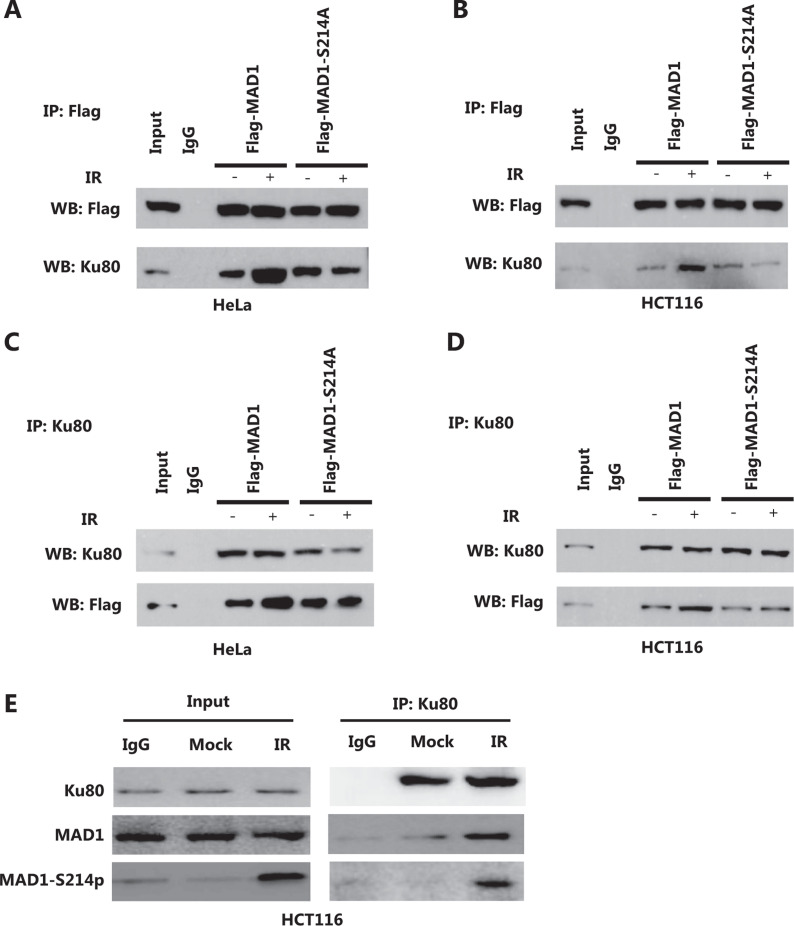
Interaction of mitotic arrest-deficient protein 1 (MAD1) with KU80 is enhanced in response to ionizing radiation (IR). FLAG-tagged wild type or S214A MAD1 was transiently transfected into HeLa or HCT116 cells. At 36 h after transfection, the cells were mock-treated or treated with IR (6 Gy). Two hours after IR, the cells were harvested and subjected to immunoprecipitation using the anti-FLAG (A and B) or anti-KU80 (C and D) antibody. The immunoprecipitates were then blotted with the indicated antibodies. (E) Co-immunoprecipitation using the anti-KU80 antibody in HCT116 cells was performed, followed by immunoblotting with the indicated antibodies.

To prioritize the validation process of the interaction, we focused on proteins that were involved in the DDR. Among the identified proteins, KU80 showed a significant increase in its interaction with MAD1 after IR in both HeLa and HCT116 cells (**Figure 4**). Using co-immunoprecipitation experiments in cells transiently expressing FLAG-tagged MAD1 (wild type or S214A), we found that in nonirradiated cells, MAD1 interaction with KU80 was not affected by the S214A mutation (**Figure 4A and 4B**), suggesting that serine 214 was not an essential site for the KU80-MAD1 interaction. However, there was more KU80 present in the FLAG precipitates after IR when compared to mock-treated cells, and we did not observe an increase in S214A mutant precipitates (**Figure 4A and 4B**). A reciprocal experiment conducted by immunoprecipitating KU80 also showed a similar effect, because the IR-induced MAD1-KU80 interaction was diminished when serine 214 was mutated to alanine (**Figure 4C and 4D**). We also pulled down KU80 and tested the MAD1 and p-MAD1 in HCT116 cells by co-immunoprecipitation experiments. As shown in **Figure 4E**, the interaction between MAD1 or p-MAD1 and KU80 increased after IR. Together, these results indicated that serine 214 phosphorylation of MAD1 was required for the IR-induced MAD1 interaction with KU80.

### IR-induced, ATM kinase-mediated MAD1 interaction with KU80 is required for DNA-PKcs activation

**Figure 5:**
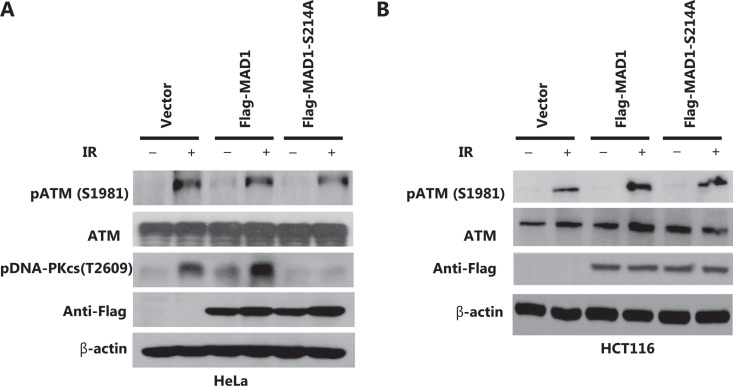
Ionizing radiation (IR)-induced mitotic arrest-deficient protein 1 (MAD1)-Ku80 interaction is required for DNA-PKcs activation. (A) HeLa cells transiently transfected with the vector control, FLAG-tagged wild type or S214A MAD1 were subjected to mock treatment or IR (6 Gy). Two hours after IR, total cell lysates were harvested and subjected to immunoblotting using the indicated antibodies. (B) HCT116 cells transiently transfected with vector control, FLAG-tagged wild type or S214A MAD1 were subjected to mock treatment or IR (6 Gy). Two hours after IR, total cell lysates were harvested and subjected to immunoblotting using the indicated antibodies.

Because KU70 and KU80 form a complex with the DNA-PKcs, which is required for non-homologous end joining repair (NHEJ), we tested whether IR-induced DNA PK activation (as measured by threonine 2609 autophosphorylation) was affected by MAD1 serine 214 phosphorylation. In HeLa cells expressing vector or wild type MAD1, IR-induced DNA-PKcs T2609 phosphorylation was observed. However, in cells expressing the S214A mutant, DNA-PKcs T2609 phosphorylation was diminished (**Figure 5A**), indicating that the MAD1 serine 214 phosphorylation dependent MAD1 interaction with KU80 was required for DNK-PKcs activation in response to IR. It should be noted that ATM kinase activation, measured by ATM kinase serine 1981 autophosphorylation in HeLa and HCT116 cells, was not affected by a S214A mutation (**Figure 5A and 5B**), further supporting that MAD1 serine 214 phosphorylation was a downstream event in the ATM kinase pathway.

### ATM kinase -mediated MAD1 phosphorylation promotes radioresistance ***in vivo***

**Figure 6:**
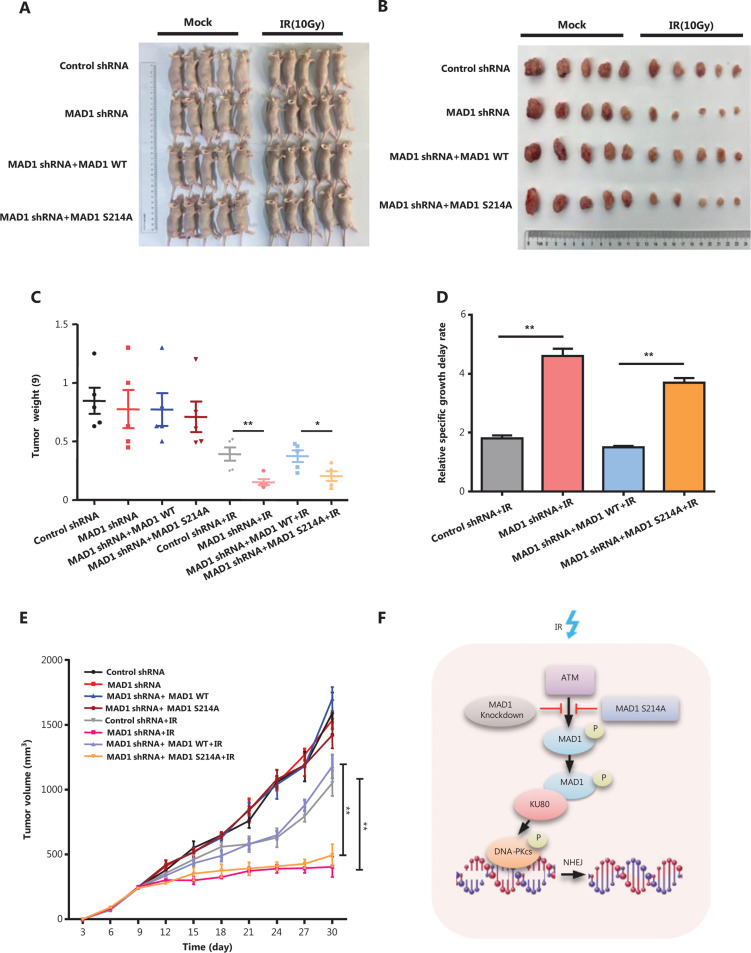
Ataxia-telangiectasia mutated (ATM) kinase-mediated mitotic arrest-deficient protein 1 (MAD1) phosphorylation promotes radioresistance *in vivo*. (A and B) BALB/c nu/nu mice bearing HCT116 xenografts. (C) The tumor weight after 30 days. (D) The relative growth delay of the tumors in response to ionizing radiation (IR). (E) Tumor growth curves after IR in xenograft mice are shown. Tumor volumes were measured every 3 days. Error bars represent the standard errors; *n* = 5. (F) The schematic model for MAD1 and serine 214 phosphorylation by ATM kinase in the DNA damage response. *P < 0.05; **P < 0.01.

Our *in vitro* data indicated that MAD1 knockdown or MAD1 S214A mutation promoted radiosensitivity. To further support this conclusion, we conducted a xenograft experiment in nude mice (**Figure 6A and 6B**). Compared with the control shRNA group or wild type group, the tumor weights in the MAD1 knockdown or the S214A mutation group was decreased after IR (**Figure 6C**). In addition, we found that the specific tumor growth delay rate significantly increased in the MAD1 knockdown group or the S214A mutation group after IR (**Figure 6D**). We also found that tumors in the MAD1 knockdown group or S214A mutated group were much more sensitive to IR than those in the control shRNA group or the wild type group (**Figure 6E**). Together, these results indicated that depleting MAD1 or mutating ATM kinase-mediated MAD1 phosphorylation promoted radiosensitivity *in vivo*.

## Discussion

Genome integrity is mostly achieved by the action of two checkpoint pathways: the DDR and SAC^1,7,8^. A prominent role of the DDR is to identify DNA damage, halt cell cycle progression, and initiate DNA repair or induce programmed cell death. The SAC is a braking system that guarantees cell cycle progression during mitosis and ensures accurate chromosome segregation. As a component of the kinetochore, MAD1 is essential for activation of the SAC^9^. MAD1 is phosphorylated by ATM kinase during mitosis in the absence of DNA damage^22^. In our study, we extended our knowledge on how the mitotic checkpoint component participates in the DDR to prevent genomic instability.

Using isogenic ATM kinase proficient/deficient cells, we showed an ATM kinase-dependent MAD1 serine 214 phosphorylation in response to IR. Notably, MAD1 serine 214 phosphorylation also caused formation of foci in the presence of DNA damage. Although the number of foci formed was less than that of γH2AX, they seem to co-localize, indicating potential recognition of double strand breaks. Although much needs to be investigated regarding the time-spatial relationship with the execution of double strand break repair, it is likely that many of the kinetochore proteins are involved in the process. This is supported by earlier studies that BUB1 and BUBR1 are involved in DNA repair^8,19–21^.

The functional significance of MAD1 serine 214 phosphorylation has been demonstrated by complementation experiments. This strong evidence prompted us to further investigate the downstream targets of ATM kinase-mediated MAD1 phosphorylation. Notably, interacting proteins with MAD1 in response to IR included many proteins regulating DNA repair and radiosensitivity. KU80 is part of the KU-heterodimer that binds to DNA double-strand break ends, which is critical for activation of NHEJ^23^. Our co-immunoprecipitation experiments confirmed that an interaction existed between MAD1 and KU80. We found that the MAD1-KU80 interaction was reduced when serine 214 was mutated to alanine, suggesting that this event may be dependent on ATM kinase-mediated MAD1 phosphorylation. The importance of the MAD1-KU80 interaction was shown by activation of DNA-PKcs (DNA-PKcs T2609 auto-phosphorylation after IR). These results further demonstrated that the signaling pathway is mediated by ATM kinase phosphorylation of MAD1 in NHEJ. However, it is still not clear how the phosphorylated form of MAD1 interacts with KU80, and how this affects the formation of the DNA-PKcs/KU70/KU80 complex.

## Conclusions

Our results showed that MAD1 serine 214 phosphorylation mediated by ATM kinase regulated DNA repair and contributed to radioresistance. Reduction of MAD1 or mutation of MAD1 S214A inhibited formation of the MAD1-KU80 complex, and decrease the DNA-PKcs threonine 2609 phosphorylation, which resulted in reduction of DNA double-strand break repair, leading to hypersensitivity to IR (**Figure 6F**). It is known that MAD1 is frequently upregulated in many tumors, such as the cervical and colorectal cancer models that we used in this study. Therefore, targeting MAD1 and/or ATM kinase-mediated MAD1 phosphorylation might represent a new strategy for cancer therapy. Furthermore, the finding that MAD1 interacted with KU80 to activate DNA-PKcs suggested that targeting the MAD1 and KU80 interaction might also be a possible approach to enhance the effectiveness of radiotherapy in these tumors.
