# Supplementary material for: Kinetochore protein MAD1 participates in the DNA damage response through ataxia-telangiectasia mutated kinase-mediated phosphorylation and enhanced interaction with KU80
Source: Cancer Biol Med. 2020 Aug 15;17(3):640–51. doi: 10.20892/j.issn.2095-3941.2020.0044 (PMC7476095; doi:10.20892/j.issn.2095-3941.2020.0044)
Supplement: Supplementary file 1 [file cbm-17-640-s001.pdf]

Supplementary materials

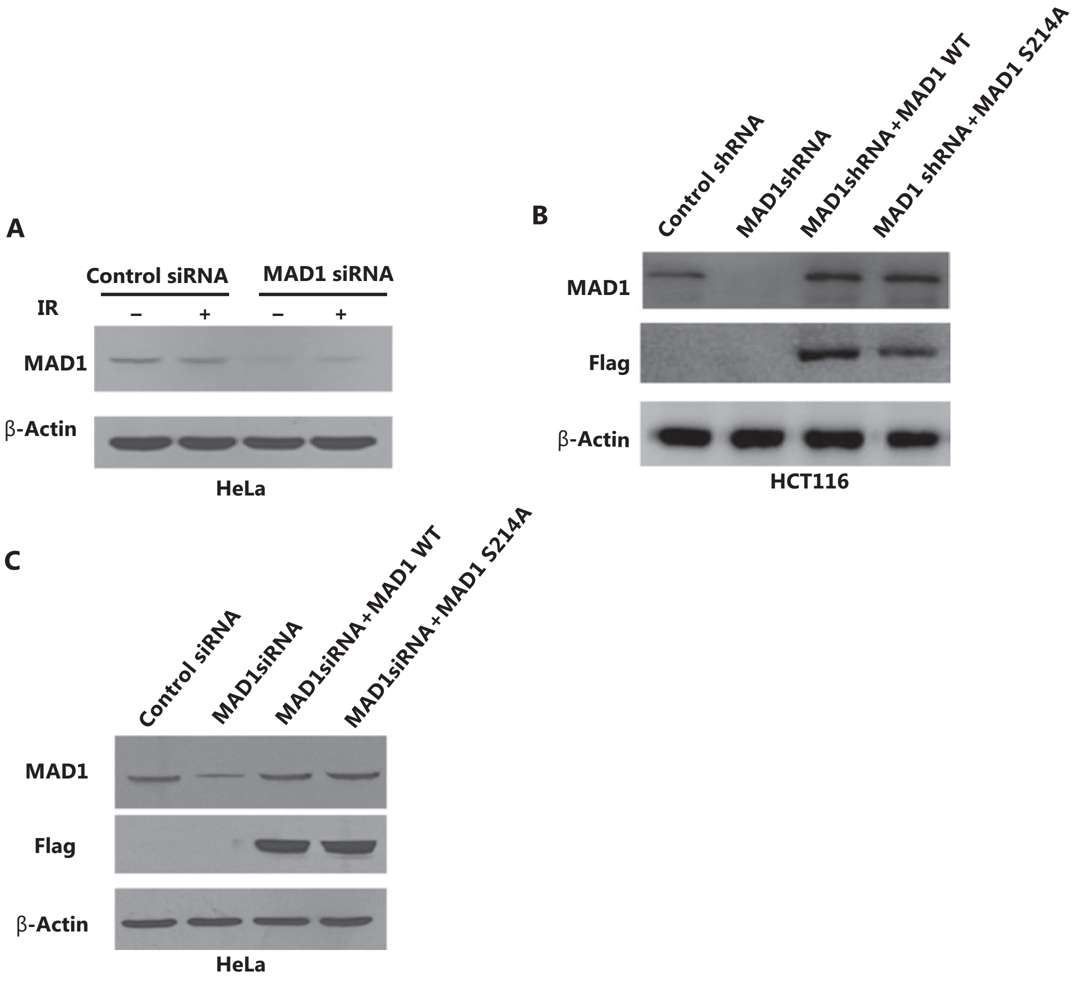

Figure S1 Isogenic cells generated in the study (A) Knockdown of mitotic arrest-deficient protein 1 (MAD1) by siRNA in HeLa cells. (B) Knockdown of MAD1 by shRNA followed by reintroduction of shRNA-resistant, FLAG-tagged wild type or S214A mutant MAD1 in HCT116 cells. (C) HeLa cells infected with siRNA against MAD1 followed by reintroduction of siRNA resistant, FLAG-tagged wild type or S214A mutant MAD1. Expression of MAD1 or FLAG-tagged MAD1 was detected by immunoblotting using the indicated antibodies.

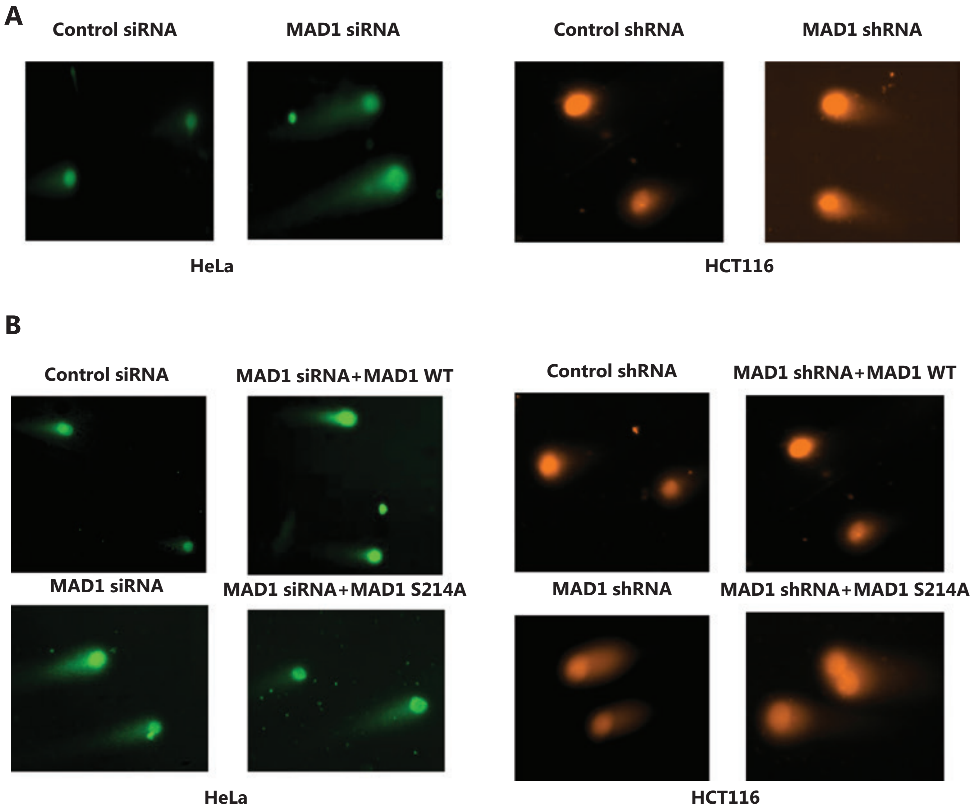

Figure S2 The single cell gel electrophoresis assay was conducted in HeLa cells (A) and HCT116 cells (B). Shown are representative pictures of comet tail formation in isogenic cell lines outlined in Supplemental Figure S1.

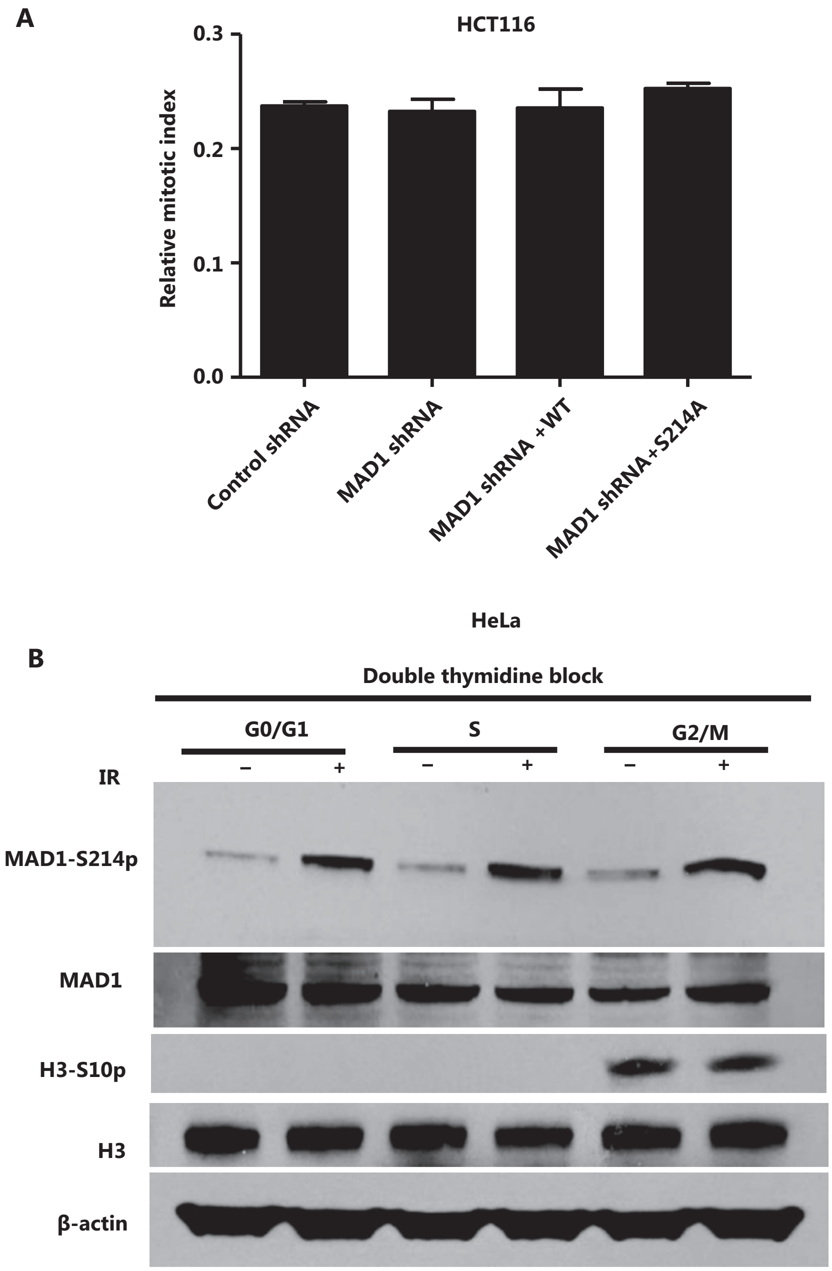

Figure S3 Ionizing radiation-induced mitotic arrest-deficient protein 1 (MAD1) serine 214 phosphorylation is independent of cell cycle stages. (A) Measurement of G2 to M transition using phosphor-histone H3 and propidium iodide staining followed by flow cytometry in HCT116 cells. (B) HeLa cells were synchronized via double thymidine block, irradiated, and measured by immunoblotting using the phosphor-histone H3, MAD1, and phosphor-MAD1 antibodies.

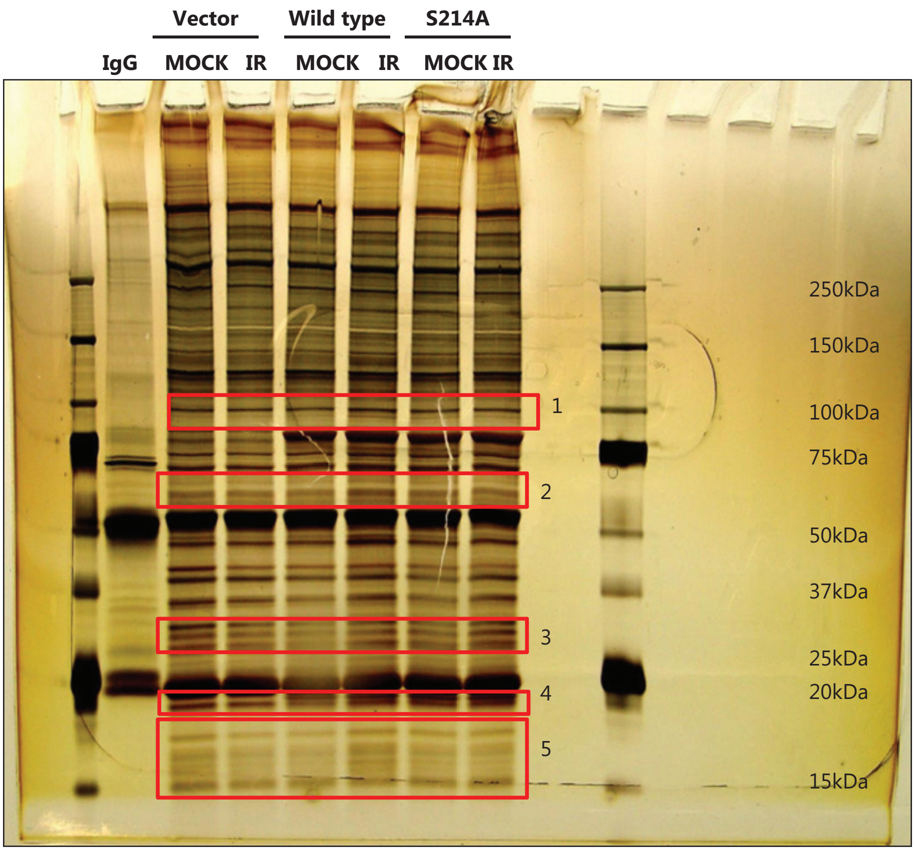

Figure S4 SDS-PAGE separation of mitotic arrest-deficient protein 1 (MAD1) immunoprecipitates. The gel was visualized using silver staining. As shown in the red frame, vector, MAD1 wild type, and MAD1 S214A transfected HeLa cells showed changes of interacting proteins. These bands were then removed and identified by mass spectrometry.

Table 1 Mass spectrometry screening results: Mitotic arrest-deficient protein 1 interacting proteins after irradiation

Band name	Molecular name (UnitProt)	Molecular weight (kDa)
1	PSMD2	100.2
	EEF2	95.3
	MCM6	92.9
	MCM3	91
	VCP	89.3
	NSUN2	86.4
2	ILF3	95.3
	TER ATPase	89.3
	HSP90AB1	83.2
	HSP90AA1	84.6
	XRCC5	82.7
	CAPRIN1	78.4
3	1433E	29.2
	1433G	28.3
	1433F	28.2
	1433B	28
	1433T	27.8
	1433Z	27.7
4	PSMB1	26.5
	PSMB6	25.4
	EIF3K	25
	MAD2L1	23.5
	PSMB3	22.9
	PRDX1	22.1
	PEBP1	21.1
5	CFL1	18.5
	NCBP2	18
	POLR2H	17.1
	EIF5A	16.8
	SUMO2	10.9
	SUB1	14.4
	NCBP2	18
	UBE2N	17.1
	DSTN	18.5

Continued

Band name	Molecular name (UnitProt)	Molecular weight (kDa)
	H3F3C	15.2
	HIST1H2AA	14.2
	POLR2H	17.1
	EIF5A	16.8
	SUMO2	10.9
	SUB1	14.4
	NCBP2	18

PSMD2, 26S proteasome non-ATPase regulatory subunit 2; EEF2, Elongation factor 2; MCM6, DNA replication licensing factor MCM6; MCM3, DNA replication licensing factor MCM3; VCP, Transitional endoplasmic reticulum ATPase; NSUN2, RNA cytosine C(5)-methyltransferase NSUN2; ILF3, Interleukin enhancer-binding factor 3; TER ATPase, Transitional endoplasmic reticulum ATPase; HSP90AB1, Heat shock protein HSP 90-beta; HSP90AA1, Heat shock protein HSP 90-alpha; XRCC5, X-ray repair cross-complementing protein 5; CAPRIN1, Caprin-1; 1433E, 14-3-3 protein epsilon; 1433G, 14-3-3 protein gamma; 1433F, 14-3-3 protein eta; 1433B, 14-3-3 protein beta/alpha; 1433T, 14-3-3 protein theta; 1433Z, 14-3-3 protein zeta/delta; PSMB1, Proteasome subunit beta type-1; PSMB6, Proteasome subunit beta type-6; EIF3K, Eukaryotic translation initiation factor 3 subunit K; MAD2L1, Mitotic spindle assembly checkpoint protein MAD2A; PSMB3, Proteasome subunit beta type-3; PRDX1, Peroxiredoxin-1; PEBP1, Phosphatidylethanolamine-binding protein 1; COF1, Cofilin-1; NCBP2, Nuclear cap-binding protein subunit 2; POLR2H, DNA-directed RNA polymerases I, II, and III subunit RPABC3; IF5A1, Eukaryotic translation initiation factor 5A-1; SUMO2, Small ubiquitin-related modifier 2; TCP4, Activated RNA polymerase II transcriptional coactivator p15; NCBP2, Nuclear cap-binding protein subunit 2; UBE2N, Ubiquitin-conjugating enzyme E2 N; DEST, Destrin; H3C, Histone H3.3C; H2A1A, Histone H2A type 1-A.
